# Camphor-Based CVD Bilayer Graphene/Si Heterostructures for Self-Powered and Broadband Photodetection

**DOI:** 10.3390/mi11090812

**Published:** 2020-08-27

**Authors:** Dung-Sheng Tsai, Ping-Yu Chiang, Meng-Lin Tsai, Wei-Chen Tu, Chi Chen, Shih-Lun Chen, Ching-Hsueh Chiu, Chen-Yu Li, Wu-Yih Uen

**Affiliations:** 1 Department of Electronic Engineering, Chung Yuan Christian University, Taoyuan City 32023, Taiwan; g10876056@cycu.edu.tw (P.-Y.C.); chrischen@cycu.edu.tw (S.-L.C.); Jimchiu@xsensetw.com (C.-H.C.); 2 Department of Materials Science and Engineering, National Taiwan University of Science and Technology, Taipei 10607, Taiwan; mltsai@gapps.ntust.edu.tw; 3 Department of Electrical Engineering, National Cheng Kung University, Tainan 70101, Taiwan; wctu@gs.ncku.edu.tw; 4 Research Center for Applied Science, Academia Sinica, Taipei 11529, Taiwan; chenchi@gate.sinica.edu.tw; 5 Xsense Technology Corp., Miaoli County 35053, Taiwan; chenyuli@xsensetw.com

**Keywords:** graphene, camphor-based CVD, self-power photodetector, graphene/Si PDs

## Abstract

This work demonstrates a self-powered and broadband photodetector using a heterojunction formed by camphor-based chemical vaper deposition (CVD) bilayer graphene on p-Si substrates. Here, graphene/p-Si heterostructures and graphene layers serve as ultra-shallow junctions for UV absorption and zero bandgap junction materials (<Si bandgap (1.1 eV)) for long-wave near-infrared (LWNIR) absorption, respectively. According to the Raman spectra and large-area (16 × 16 μm^2^) Raman mapping, a low-defect, >95% coverage bilayer and high-uniformity graphene were successfully obtained by camphor-based CVD processes. Furthermore, the carrier mobility of the camphor-based CVD bilayer graphene at room temperature is 1.8 × 10^3^ cm^2^/V·s. Due to the incorporation of camphor-based CVD graphene, the graphene/p-Si Schottky junctions show a good rectification property (rectification ratio of ~110 at ± 2 V) and good performance as a self-powered (under zero bias) photodetector from UV to LWNIR. The photocurrent to dark current ratio (PDCR) value is up to 230 at 0 V under white light illumination, and the detectivity (D*) is 8 × 10^12^ cmHz^1/2^/W at 560 nm. Furthermore, the photodetector (PD) response/decay time (i.e., rise/fall time) is ~118/120 μs. These results support the camphor-based CVD bilayer graphene/Si Schottky PDs for use in self-powered and ultra-broadband light detection in the future.

## 1. Introduction

Si-based photodetectors (PDs) are widely used in pollution analyzers, biological research, combustion flame monitoring, and optical communication due to their compatibility with the silicon integrated circuit (IC) industry [1,2,3]. Until now, Si-based PDs with different structures (e.g., Schottky photodiodes, p-n photodiodes, metal-semiconductor-metal (MSM) photodiodes, phototransistors, and p-i-n photodiodes) have been intensively studied and developed [4,5,6]. Among them, Si Schottky photodiodes provide superior radiation resistance and high-speed operation and can be easily integrated with photoelectronic and microelectromechanical systems for photon detection [7,8]. Furthermore, the self-powered operation mode (at zero bias) of Schottky photodiodes could provide many benefits for intelligent sensors working in 5th generation wireless systems [9,10]. However, conventional Si-based PDs with relatively low photoresponses in the UV (<400 nm) [2] and long-wave near-infrared (LWNIR) (>1100 nm) regions [11] are limited to visible light (400–700 nm) and short-wave near-infrared (SWNIR: 700–1100 nm) detection due to the shallow penetration depth in the UV region [12,13] and the Si transparence in the LWNIR region [2,13], respectively, leading to the restrictions on broadband spectral response. To improve the broadband detection of Si-based PDs, junction material systems consisting of an ultra-shallow junction (for UV detection) based on a narrow bandgap (<Si bandgap (1.1 eV)) material (for LWNIR detection) are required and expected to offer a high carrier separation/collection efficiency under high-speed operation. Graphene, on the other hand, is a zero bandgap and one-atom-thick material with outstanding electronic and optical properties, such as its ultra-high carrier mobility (up to 200,000 cm^2^/Vs), ultra-broadband absorption from UV to THz, and ultra-fast luminescence (in picosecond scale) [14,15,16,17]. Thus, graphene is viewed as an ideal material to replace metal contacts and transparent conducting oxide (TCO) electrodes (e.g., indium tin oxide (ITO), indium zinc oxide (IZO), and fluorine-doped tin oxide (FTO)) in optoelectronic devices for forming ultra-shallow junctions with ultra-broadband optical absorption [18,19,20]. After the first graphene/Si Schottky PDs reported by M. Amirmazlaghani et al. [21], the fast development of graphene/Si Schottky PDs is targeted specifically at the enhancement of the responsivity by employing Si nanostructures and chemical doping, as well as metal nanoparticles (such as Au and Pt) [22,23,24,25]. However, the complex, high-cost, and environmentally unfriendly processes of the above-mentioned methods have hindered the practical applications of graphene/Si PDs and integration with complementary metal–oxide–semiconductor (CMOS) processes.

Among the conventional synthetic methods for graphene, chemical vaper deposition (CVD) is viewed as the most important means for the production of wafer-scale graphene with a high uniformity and low amount of defects, as well as a controlled layer number and morphology, which has gathered much attention since it was reported in 2008 and 2009 [26,27]. In the CVD synthesis process, carbon sources (such as CH_4_ and C_2_H_2_) decompose to carbon atoms or carbon radicals and then rearrange into a graphene layer on the catalytic metal (e.g., Ni and Cu) surface at a high temperature (900~1000 °C) [28]. However, the purified carbon gaseous sources (such as CH_4_ and C_2_H_2_) of all CVD methods are expensive and environmentally unfriendly, obstructing the development of graphene-based devices for practical applications of optoelectronics [29]. It should be noted that CH_4_ is a strong greenhouse gas (about 28 times stronger than CO_2_ at warming the Earth) [30]. As compared with the CVD graphene from conventional hydrocarbon gas precursors in previous several reports, camphor can be better for catalytic decomposition in the CVD process of graphene synthesis. This is due to the hexagonal carbon rings and hydrocarbons of the camphor molecular structure for good coordination during the graphene formation [31]. Furthermore, natural camphor exhibits inexpensive and environmentally friendly characteristics as the carbon source in the CVD processes. In 2006, nano-graphene synthesized using camphor-based CVD was reported by Somani et al. However, the nano-scale, thick (~35 layers), and disordered layer structures limit the applications of camphor-based CVD nano-graphene [32]. In 2012, several-layer (~5 layers) graphene synthesized on Cu foil using camphor in a surface wave plasma CVD system was reported by Kalita et al. [31]. Furthermore, bi/several-layer graphene production on Ni foil by the dissociation of camphor molecules in CVD processes was demonstrated by Ravani et al. in 2013 [33]. However, the graphene mentioned above still suffered from a small size (1~2 μm) and low uniformity. Very recently, camphor-based graphene grown on Cu foil with a large area size was obtained, but its carrier mobility was not high (≤10^3^ cm^2^/V·s) [34]. Despite the several investigations of the synthesis of graphene layers by natural camphor, more practical applications are required in order to prove their feasibility.

In this letter, a camphor-based CVD bilayer graphene/Si Schottky PD with self-powered and broadband detection was demonstrated. The graphene not only cooperates with p-Si to form an ultra-shallow junction for the enhancement of UV but also improves LWNIR absorption because of its zero bandgap. From Raman spectra analyses, large-area Raman mapping, and Hall measurements (at room temperature), the graphene grown by camphor-based CVD exhibits few defects, >95% coverage bilayer structures, a high-uniformity, and a high carrier mobility (1.8 × 10^3^ cm^2^/V·s). The rectification ratio of the camphor-based CVD bilayer graphene/Si Schottky PD is ~110 at ±2 V. The camphor-based CVD bilayer graphene/Si Schottky PDs in this study show ultra-broadband (from UV to LWNIR) photoresponsivity, a high photocurrent-to-dark-current ratio (up to 230), and a high detectivity (D*) (8 × 10^12^ cmHz^1/2^/W at 560 nm) under zero bias. Moreover, the camphor-based CVD bilayer graphene/Si Schottky PDs also exhibits a fast photoresponse property (rise/fall time ~118/120 μs). This research could provide a new means of low-cost production of the camphor-based CVD bilayer graphene/Si Schottky PDs and extend the applications of Si-based devices.

## 2. Device Fabrication and Methods

**Figure 1:**
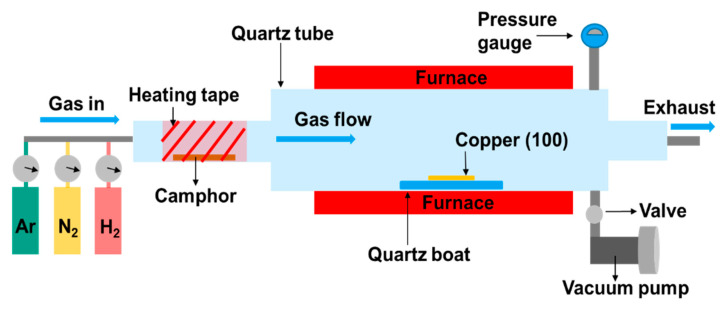
Schematic diagram of our atmospheric pressure chemical vapor deposition (APCVD) setup.

**Figure 2:**
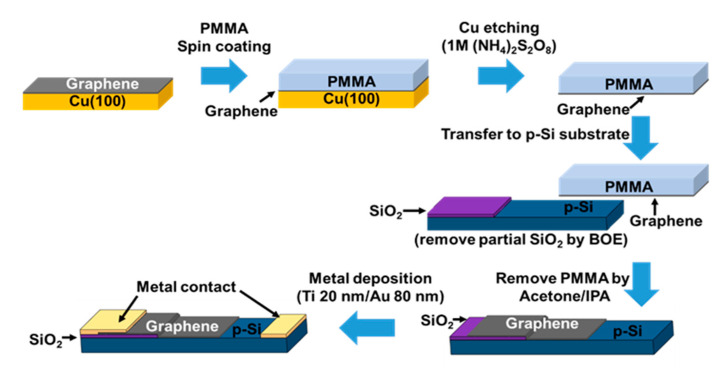
Process flow diagram of the camphor-based chemical vaper deposition (CVD) bilayer graphene/p-Si photodetector (PD) that was fabricated.

A copper (100) substrate (thickness: 0.3 mm, the Nilaco corporation) was cleaned by acetone for 3 min in the ultrasonic cleaner. After that, the copper substrate was blow dried by N_2_ gas and then placed on the middle of in the reactor (a 1.2 m-length quartz tube with a 1-inch diameter). For the synthesis of graphene, the copper substrate was annealed in Ar (with flow rate: 200 sccm) and H_2_ (with flow rate: 100 sccm) in ambient conditions under 760 torr at 950 °C for 80 min and 15 min, respectively. Then, 1.2 mg of camphor powder (Yu Li Hang biochemical industrial co.) was heated at 125 °C, and its vapors were sent into the reactor by carrier gas (Ar/H_2_ flow rate: 98/8 sccm) under 760 torr for 10 min. As shown in Figure 1, the schematic diagram is our atmospheric pressure chemical vapor deposition (APCVD) system. After the process of growing graphene on copper substrate, the graphene layers were transferred to p-type Si (111) substrates (resistivity of 1–30 Ω cm), with the top SiO_2_ film being partially removed (etched by buffered oxide etchant (BOE) (HF:NH_4_F = 1:5)) and cleaned in acetone and isopropanol prior to use. Figure 2 is the schematic of the graphene transfer and PD fabrication process flow.

The morphology of the graphene/Si heterostructure was analyzed using a field emission scanning electron microscope (FESEM). The characteristics and dimensions of the graphene layers were confirmed by micro Raman spectroscopy (the resolution ~1.4 cm^−1^) with 532 nm laser excitation. The Hall mobility of the camphor-based CVD graphene was measured with an Ecopia HMS-5000 (Ecopia corporation, Anyang, South Korea) at room temperature.

The Ohmic contacts of the camphor-based CVD bilayer graphene/Si heterojunction (active region: 9 mm × 8 mm) were deposited with Ti (20 nm)/Au (80 nm) using the thermal evaporator. Photocurrent was generated under the illumination of solar simulator (LSH 150, Taiwan Fiber Optics, Inc., Taipei, Taiwan). The spectral responsivity of the camphor-based CVD bilayer graphene/Si PDs was measured with the QEX10—Quantum Efficiency Measurement System (PV Measurements, Inc., Boulder, CO, USA—under zero bias. The *I-V* characteristics of the fabricated PDs were measured by an Agilent (B1500 A) source meter (Keysight Technologies, Santa Rosa, CA, USA) The photocurrent and dark current as a function of time were measured by a Keithley 2612B (Keithley Instruments, Cleveland, OH, USA) source meter with a chopper to switch on/off the white light.

## 3. Measurement Results and Discussion

**Figure 3:**
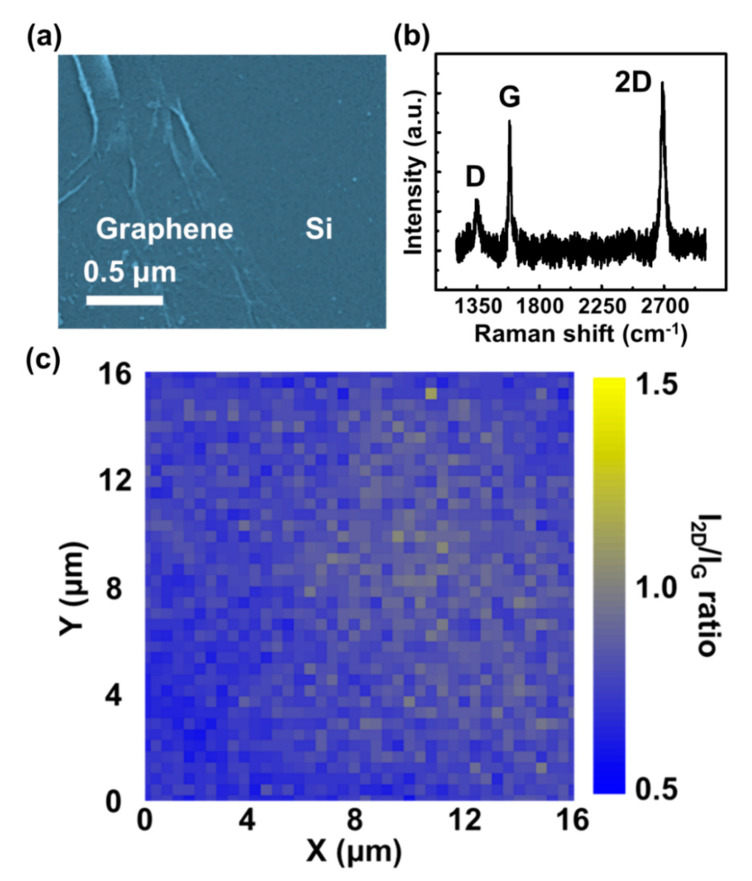
(**a**) Field emission scanning electron microscope (FESEM) image and (**b**) Raman spectra and (**c**) *I_*2*D_/I_G_* Raman mapping of the bilayer graphene on a p-Si substrate (excitation laser:532 nm).

The FESEM image of the morphology of graphene/Si heterostructure is shown in Figure 3a. There are no obvious cracks and holes in the graphene area after optimizing the transfer processes. Furthermore, some wrinkles of graphene are also observed, induced by the solution interface and the defects of the Cu substrate during the transfer processes [35]. In order to realize the quality and the layer number of the camphor-based CVD graphene in this study, the Raman spectra are shown in Figure 3b; the peak positions for the D band, G band, and 2D band in the graphene are 1350, 1600, and 2700 cm^−1^, respectively [36,37]. The intensity ratio of the 2D and G peaks (i.e., *I_*2*D_/I_G_*) and the full width at half maximum (FWHM) of the 2D peak can be used to further determine the graphene layer number (i.e., *I_*2*D_/I_G_* > 1.3 and FWHM < 30 cm^−1^ representing single-layer graphene and *I_*2*D_/I_G_* < 0.7 and FWHM > 70 cm^−1^ representing three or more layers of graphene) [36,37,38,39,40]. The calculated *I_*2*D_/I_G_* ratio value and FWHM of the 2D peak are ~1 and 45 cm^–1^, respectively, corresponding to bilayer graphene. However, the weak D peak indicates that the few defects in the graphene layers could be induced during high-temperature CVD processes. Two-dimensional Raman mapping is used to investigate the uniformity of graphene across the surface. In Figure 3c, the Raman mapping for the camphor-based CVD graphene shows the variation in the *I_*2*D_*/*I_G_* ratio at each sampling point (each sampling step: 400 nm) over a 16 μm × 16 μm region, indicating the presence of uniform bilayer graphene with a high quality and high coverage throughout the surface [36,37]. For semiconductor materials, carrier mobility is a critical parameter for determining the performance of optoelectronic devices. Therefore, the carrier mobility (1.8 × 10^3^ cm^2^/V·s) of the camphor-based CVD bilayer graphene was obtained from the room temperature Hall measurement. Compared to the conventional CVD graphene (mobility: 100~1000 cm^2^/V·s) [41,42], the camphor-based CVD graphene shows a higher mobility. However, the biggest issue of CVD graphene is that its carrier mobility is much lower than that of graphene obtained from the exfoliation of graphite.

**Figure 4:**
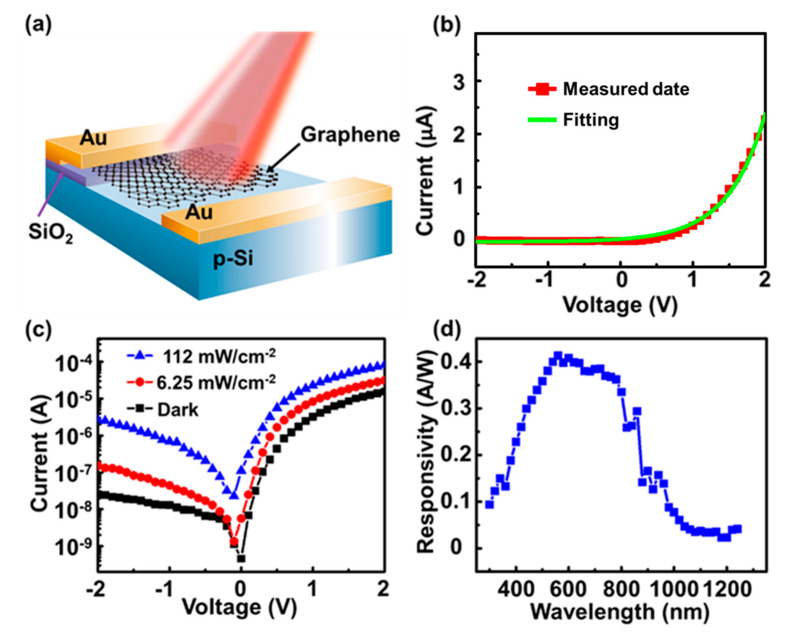
(**a**) Schematic of the camphor-based CVD bilayer graphene/p-Si Schottky PD; (**b**) *I-V* and fitting curves of the camphor-based CVD bilayer graphene/p-Si Schottky PDs in the dark; (**c**) *I-V* curves of the camphor-based CVD bilayer graphene/p-Si Schottky PDs in the dark and under white light illumination with 6.25 and 112 mW/cm^−2^, respectively. (**d**) Spectral responsivity of the camphor-based CVD bilayer graphene/p-Si Schottky PDs under zero bias.

In the schematic of the camphor-based CVD bilayer graphene/p-Si Schottky PDs, as shown in Figure 4a, the graphene/p-Si junction area (i.e., active region) is ~9 mm × 8 mm and the thickness of the metal contact and SiO_2_ are 100 nm. Figure 4b shows the *I-V* and fitting curves of the camphor-based CVD bilayer graphene/p-Si Schottky PDs measured with an Agilent (B1500 A) source meter in the dark. The reverse dark current and a rectification ratio of the camphor-based CVD bilayer graphene/p-Si Schottky PD are ~20 nA (@−2 V bias) and ~110 at ±2 V bias, respectively, indicating the good rectification property of the camphor-based CVD graphene/p-Si Schottky junction even with a bilayer-graphene thickness as low as ~0.7 nm [43]. Furthermore, the rectification *I-V* characteristics can be clarified by using *I* = AA* *T*^2^ exp[−qΦ_B_/*k_B_T*] [exp(*qV*/*nk_B_T*)−1] based on thermionic emission theory [44,45], where *A* is the effective graphene/p-Si junction area (0.72 cm^2^), A* is the Richardson constant (≈32 A cm^−2^ K^−2^ for p-Si) [46], *T* is the temperature (300 K), *q* is the electron charge (1.6 × 10^−19^ C), qΦ_B_ is the Schottky barrier high (SBH) of the graphene/p-Si Schottky junction, n is the ideal factor(~4.5), and *k_B_* is the Boltzmann constant. Therefore, the SBH (~0.81 eV) in this study could be extracted further by fitting the rectification *I-V* curve using the above equation. One should note that ideal factor value is >1, indicating that the inhomogeneity (resulting from surface roughness or defects) in the graphene/p-Si Schottky junction area [46].

**Figure 5:**
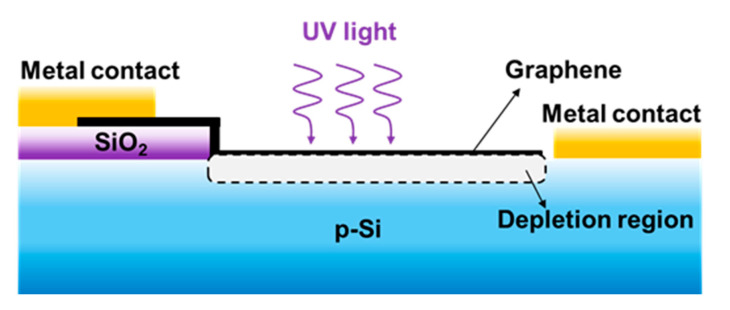
Schematic cross-section of the camphor-based CVD bilayer graphene/p-Si Schottky PD structure.

Figure 4c shows the *I-V* characteristics of the camphor-based CVD bilayer graphene/p-Si Schottky PDs measured in the dark and under white light illumination of different power densities. At zero bias, the photocurrent to dark current ratio (PDCR = (*I_p_* − *I_d_*)/*I_d_*, where *I_d_* is the dark current and *I_p_* is the photocurrent) [47] of the camphor-based CVD graphene/p-Si Schottky PDs is 13.25 and 230 under white light illumination, with 6.25 and 112 mW/cm^−2^, respectively. From the PDCR values, the camphor-based CVD bilayer graphene/p-Si Schottky PDs are still available even under zero bias, mainly owing to the photovoltaic properties of Schottky junctions [48,49]. To demonstrate the feasibility of camphor-based CVD bilayer graphene/p-Si Schottky PDs in practical uses, a spectral responsivity analysis was performed under zero bias from UV to LWNIR (300 to 1250 nm), as shown in Figure 4d. The responsivity is 0.09 A/W at 300 nm (UV region), 0.41 A/W at 560 nm (visible light region), and 0.04 A/W at 1250 nm (LWNIR region), indicating that the ultra-broadband detection of the camphor-based CVD bilayer graphene/p-Si Schottky PDs is owing to the effective UV light absorption of the ultra-shallow graphene/p-Si Schottky junction and the LWNIR absorption (>Si cut-off wavelength) of graphene (zero bandgap). As compared with commercial Si PN and PIN PDs [20], the depletion region of the ultra-shallow junction will be formed near the top surface of the camphor-based CVD bilayer graphene/p-Si Schottky PDs, as shown in Figure 5. This would lead to the direct absorption of UV light therein, without a long penetration distance. The depletion region width is evaluated to be ~0.18 μm using *W* = [2*ε*_0_*ε_r_* (Φ_bi_ + *V*)/*eN_d_*]^1/2^ [44], where the free space permittivity is *ε_0_* = 8.85 × 10^−14^ F/cm, the relative permittivity of Si is *ε_r_* = 11.8, the built-in potential is Φ_bi_ = ~0.62 V (Appendix A), the reverse-bias voltage is *V* = 0 V, and the doping concentration is *N_d_* = ~2 × 10^16^ cm^−3^. Thus, the photocarriers could be effectively induced by UV light due to a limited UV penetration depth (~20 nm) within the depletion region formed [20].

Note that the responsivity obtained here is competitive with that obtained from the conventional CVD graphene/Si Schottky PDs (0.51 A/W at 532 nm) [49] and much higher than that obtained from the pristine graphene PDs (1 × 10^−3^ A/W) [47], graphene oxide/Si Schottky PDs (~63 mA/W at 445 nm), [50] and graphene/insulator/Si (GIS) PDs (~20 mA/W at 1200 nm) [8]. Furthermore, the detectivity (D*) is an important parameter used to analyze the PD performance [51,52]. Under zero bias, the detectivity (D*) of the camphor-based CVD graphene/p-Si Schottky PDs at 560 nm in this study is 8 × 10^12^ cmHz^1/2^/W by D* = (*f*^1/2^)/*P* = *R*/(2*eJ_d_*)^1/2^, where *P* is the incident optical power, *R* is the responsivity, *e* is 1.6 × 10^−19^ C, *J_d_* is the dark current density, and f is the frequency bandwidth of the PD [47,51]. Compared with graphene oxide/Si Schottky PDs (~1.2 × 10^12^ cm Hz^1/2^/W) [50] and the conventional CVD graphene/Si PDs (~10^10^ cm Hz^1/2^/W) [53], the detectivity of the camphor-based CVD graphene/p-Si Schottky PDs exhibits a better performance.

**Figure 6:**
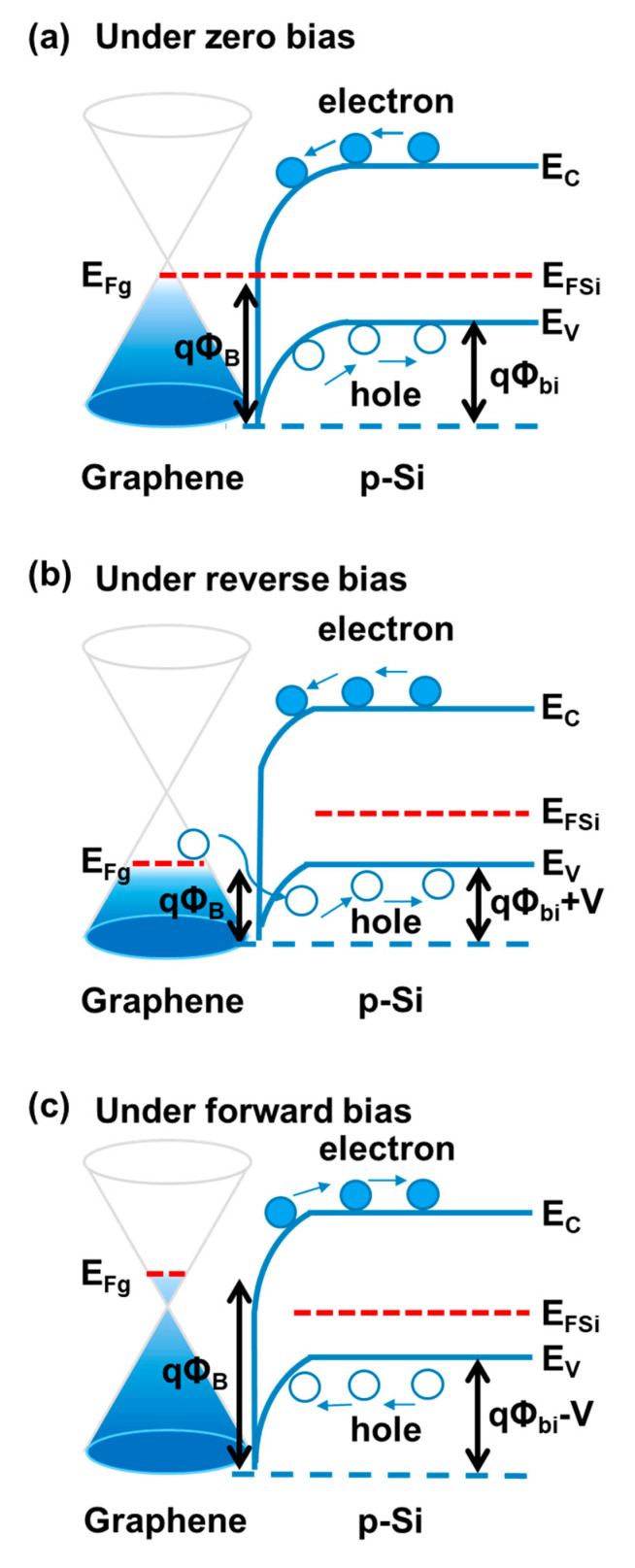
Band diagram of the camphor-based CVD bilayer graphene/p-Si PDs, where E_Fg_, E_FSi_, qΦ_B_ (~0.81 eV), and qΦ_bi_ (~0.62 eV) are the Fermi energy of graphene, the Fermi energy of Si, the Schottky barrier height (SBH), and the hole barrier height, respectively. (**a**) Under zero bias, (**b**) under reverse bias, and (**c**) under forward bias. Charge carriers (holes) are shown as open circles.

As shown in Figure 6, the band structures based on Anderson’s rule are used to clarify the physics behind the above-observed *I-V* characteristics of camphor-based CVD graphene/p-Si Schottky PDs [46,54,55,56,57]. Figure 6a is the band structure of the camphor-based CVD graphene/p-Si heterostructure at thermal equilibrium under zero bias. As the reverse bias applied to the camphor-based CVD graphene/p-Si PDs (i.e., graphene is at a positive voltage compared to p-Si), the graphene Fermi level (E_Fg_) is lower than the Si Fermi energy (E_FSi_), leading to the SBH (qΦ_B_) lowering, as shown in Figure 6b. In the beginning, the hole injection will be inhibited by the Si bandgap, and then it increases gradually with increasing reverse bias because of the SBH decrease. While under forward bias as shown in Figure 6c, the E_Fg_ is higher than E_FSi_ (i.e., graphene is at a negative voltage compared to p-Si), resulting in the increase in SBH. Moreover, the barrier height (qΦ_bi_) of the hole from p-Si to graphene will be reduced gradually with an increasing forward bias, leading to a higher dark current. Therefore, the dark current under a low reverse bias (from 0 V to −2 V) is much lower than under a low forward bias (form 0 V to +2 V), as shown in Figure 4b,c. It is noteworthy that the SBH tunable behavior of the camphor-based CVD graphene/p-Si heterostructure is consistent with the numerous study results of the conventional CVD bilayer graphene/Si heterostructures reported previously [58,59,60,61,62,63].

**Figure 7:**
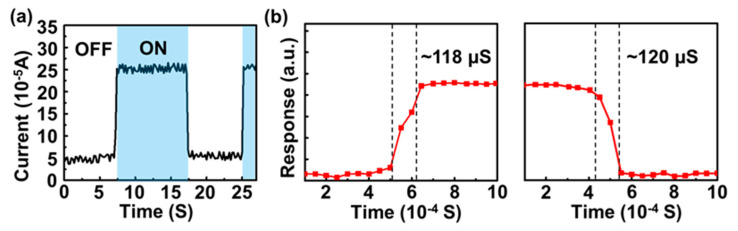
(**a**) The photocurrent and dark current as a function of time of the prepared bilayer graphene/p-Si Schottky PD under a 3 V bias. (**b**) High-resolution time response of the prepared bilayer graphene/p-Si Schottky PD.

To highlight the stable and high-speed photoresponse properties of the camphor-based CVD bilayer graphene/p-Si Schottky PDs, the time-resolved measurements were performed under a 3 V bias, as shown in Figure 7a. The current increased to a high value (i.e., ON state) and then decreased to a low value (i.e., OFF state) with switching on and off the light, respectively; the stable and reversible photoresponse was also observed even with a small PDCR value (~4) under 3 V bias. Note that the relatively low PDCR value is possibly due to the increase in the dark current under forward bias. Furthermore, in order to quantize the PD operation speed, the rise/fall time of PDs can be revealed by the transition between the light ON and OFF states. As shown in Figure 7b, the rise time (the time difference from 10% to 90% of the saturation photocurrent after switching on the light) and the fall time (the time difference from 90% to 10% of the saturation photocurrent after switching off the light) of the camphor-based CVD bilayer graphene/p-Si Schottky PDs can be revealed to be 118 and 120 μs, respectively [64,65,66,67]. Compared with conventional CVD graphene/Si PDs [49,53], conventional CVD graphene/ZnO PDs [68], and graphene oxide/Si Schottky PDs [50], the camphor-based CVD bilayer graphene/p-Si PDs show a much faster operation speed, resulting from the higher carrier mobility of camphor-based CVD graphene than the conventional CVD graphene and better graphene/p-Si junction quality.

## 4. Conclusions

In summary, a self-powered and broadband PD using a camphor-based CVD bilayer graphene/p-Si heterojunction was fabricated. Here, graphene layers could unite with p-Si to form ultra-shallow Schottky junctions for absorbing UV light effectively and, at the same time, graphene layers with zero bandgap could also provide a higher LWNIR absorption than Si. Raman spectra analyses, large-area (16 × 16 μm^2^) Raman mapping images, and Hall measurements could reveal that our camphor-based CVD graphene has low defects, >95% coverage bilayer structures, a high-uniformity, and a high carrier mobility (1.8 × 10^3^ cm^2^/V·s). By employing camphor-based CVD bilayer graphene/p-Si Schottky junctions, our PDs also show a high rectification ratio value (~110 at ±2 V) and high photo-sensing performances (such as an ultra-broadband (from UV to LWNIR) photoresponsivity (under zero bias), a high PDCR value (up to 230 at 0 V), and high detectivity (D*) (8 × 10^12^ cmHz^1/2^/W at 560 nm)). In addition, the operation speed of PD was revealed by the rise time and fall time as fast as 118 and 120 μs, respectively. This work demonstrates that the low-cost camphor-based CVD bilayer graphene incorporating p-Si holds promise for practical applications in the new generation of self-powered and ultra-broadband photon detection.

## Appendix A

The barrier height (qΦ_bi_ ~ 0.62 eV) of hole could be estimated by qΦ_bi_ = qΦ_B_ − [(E_g_/2) − *k_B_T*ln(P_0_/n_i_)] [44], where qΦ_B_ is the SBH of the graphene/p-Si Schottky junction, E_g_ is the Si bandgap (1.12 eV), *k_B_* is the Boltzmann constant, *T* is the temperature (300 K), P_0_ is doping concentration (~2 × 10^16^ cm^−3^) and n_i_ is intrinsic carrier concentration (1.45 × 10^10^ cm^−^*^3^*). Therefore, the built-in potential (Φ_bi_) is ~0.62 V.

## References

[1] Monroy E., Omnes F., Calle F. (2003). Wide-bandgap semiconductor ultraviolet photodetectors. Semicond. Sci. Technol..

[2] Tsai D.-S., Lin C.-A., Lien W.-C., Chang H.-C., Wang Y.-L., He J.-H. (2011). Ultra-high-responsivity broadband detection of Si metal–semiconductor–metal Schottky photodetectors improved by ZnO nanorod arrays. ACS Nano.

[3] Wei T.-C., Tsai D.-S., Ravagger P., Ke J.-J., Tsai M.-L., Lien D.-H., Huang C.-Y., Horng R.-H., He J.-H. (2014). See-through Ga_2_O_3_ solar-blind photodetectors for use in harsh environments. IEEE J. Sel. Top. Quant. Electron..

[4] Zhang Y., Ji T., Zhang W., Guan G., Ren Q., Xu K., Huang X., Zou R., Hu J. (2017). A self-powered broadband photodetector based on an n-Si (111)/p-NiO heterojunction with high photosensitivity and enhanced external quantum efficiency. J. Mater. Chem. C.

[5] Thahe A.A., Bakhtiar H., Bidin N., Hassan Z., Qaeed M.A., Ramizy A., Talib Z.A., Ahmed N.M., Omar K., Alqaraghuli H. (2018). High-performance nanoporous silicon-based photodetectors. Optik.

[6] Virot L., Benedikovic D., Szelag B., Alonso-Ramos C., Karakus B., Hartmann J.-M., Roux X.L., Crozat P., Cassan E., Marris-Morini D. (2017). Integrated waveguide PIN photodiodes exploiting lateral Si/Ge/Si heterojunction. Opt. Express.

[7] Malinowski P.E., John J., Duboz J.Y., Hellings G., Lorenz A., Madrid J.G.R., Sturdevant C., Cheng K., Leys M., Derluyn J. (2009). Backside-illuminated GaN-on-Si Schottky photodiodes for UV radiation detection. IEEE Electron. Dev. Lett..

[8] Riazimehr S., Kataria S., Gonzalez-Medina J.M., Wagner S., Shaygan M., Suckow S., Ruiz F.G., Engstrom O., Godoy A., Lemme M.C. (2019). High responsivity and quantum efficiency of graphene/silicon photodiodes achieved by interdigitating Schottky and gated regions. ACS Photonics.

[9] Wen F., Wang H., He T., Shi Q., Sun Z., Zhu M., Zhang Z., Cao Z., Dai Y., Zhang T. (2020). Battery-free short-range self-powered wireless sensor network (SS-WSN) using TENG based direct sensory transmission (TDST) mechanism. Nano Energy.

[10] Dong B., Yang Y., Shi Q., Xu S., Sun Z., Zhu S., Zhang Z., Kwong D.-L., Zhou G., Ang K.-W. (2020). Wearable triboelectric−human−machine interface (THMI) using robust nanophotonic readout. ACS Nano.

[11] Kim K., Yoon S., Seo M., Lee S., Cho H., Meyyappan M., Baek C.-K. (2019). Whispering gallery modes enhance the near-infrared photoresponse of hourglass-shaped silicon nanowire photodiodes. Nat. Electron..

[12] Shi L., Nihtianov S. (2012). Comparative study of silicon-based ultraviolet photodetectors. IEEE Sens. J..

[13] Xie C., Lu X.T., Tong X.W., Zhang Z.X., Liang F.X., Liang L., Luo L.B., Wu Y.C. (2019). Recent progress in solar-blind deep-ultraviolet photodetectors based on inorganic ultrawide bandgap semiconductors. Adv. Funct. Mater..

[14] Geim A.K., Novoselov K.S. (2007). The rise of graphene. Nat. Mater..

[15] Goykhman I., Sassi U., Desiatov B., Mazurski N., Milana S., Fazio D., Eiden A., Khurgin J., Shappir J., Levy U. (2016). On-chip integrated, silicon–graphene plasmonic schottky photodetector with high responsivity and avalanche photogain. Nano Lett..

[16] Yan W., Harley-Trochimczyk A., Long H., Chan L., Pham T., Hu M., Qin Y., Zettl A., Carraro C., Worsley M.A. (2017). Conductometric gas sensing behavior of WS_2_ aerogel. FlatChem.

[17] Ogawa S., Shimatani M., Fukushima S., Okuda S., Kanai Y., Ono T., Matsumoto K. (2019). Broadband photoresponse of graphene photodetector from visible to long-wavelength infrared wavelengths. Opt. Eng..

[18] Savchak M., Borodinov N., Burtovyy R., Anayee M., Hu K., Ma R., Grant A., Li H., Cutshall D.B., Wen Y. (2018). Highly conductive and transparent reduced graphene oxide nanoscale films via thermal conversion of polymer-encapsulated graphene oxide sheets. ACS Appl. Mater. Interfaces.

[19] Ma Y., Zhi L. (2019). Graphene-based transparent conductive films: Material systems, preparation and applications. Small Methods.

[20] Wan X., Xu Y., Guo H., Shehzad K., Ali A., Liu Y., Yang J., Dai D., Lin C.-T., Liwei L. (2017). A self-powered high-performance graphene/silicon ultraviolet photodetector with ultra-shallow junction: Breaking the limit of silicon?. NPJ 2D Mater. Appl..

[21] Amirmazlaghani M., Raissi F., Habibpour O., Vukusic J., Stake J. (2013). Graphene-Si schottky IR detector. IEEE J. Quant. Electron..

[22] Kim J., Joo S.S., Lee K.W., Kim J.H., Shin D.H., Kim S., Choi S.-H. (2014). Near-ultraviolet-sensitive graphene/porous silicon photodetectors. ACS Appl. Mater. Interfaces.

[23] Luo L.-B., Zeng L.-H., Xie C., Yu Y.-Q., Liang F.-X., Wu C.-Y., Wang L., Hu J.-G. (2014). Light trapping and surface plasmon enhanced high-performance NIR photodetector. Sci. Rep..

[24] Huang K., Yan Y., Li K., Khan A., Zhang H., Pi X., Yu X., Yang D. (2017). High and fast response of a graphene–silicon photodetector coupled with 2D fractal platinum nanoparticles. Adv. Opt. Mater..

[25] Shin D.H., Choi S.-H. (2018). Graphene-based semiconductor heterostructures for photodetectors. Micromachines.

[26] Yu Q., Lian J., Siriponglert S., Li H., Chen Y.P., Pei S.-S. (2008). Graphene segregated on Ni surfaces and transferred to insulators. Appl. Phys. Lett..

[27] Kim K.S., Zhao Y., Jang H., Lee S.Y., Kim J.M., Kim K.S., Ahn J.-H., Kim P., Choi J.-Y., Hong B.H. (2009). Large-scale pattern growth of graphene films for stretchable transparent electrodes. Nature.

[28] Khan A., Islam S.M., Ahmed S., Kumar R.R., Habib M.R., Huang K., Hu M., Yu X., Yang D. (2018). Direct CVD growth of graphene on technologically important dielectric and semiconducting substrates. Adv Sci..

[29] Kwon S.-J., Seo H.-K., Ahn S., Lee T.-W. (2019). Value-added recycling of inexpensive carbon sources to graphene and carbon nanotubes. Adv. Sustain. Syst..

[30] Nikolay V., Balashov N.V., Davis K.J., Miles N.L., Lauvaux T., Richardson S.J., Barkley Z.R., Bonin T.A. (2020). Background heterogeneity and other uncertainties in estimating urban methane flux: Results from the Indianapolis Flux Experiment (INFLUX). Atmos. Chem. Phys..

[31] Kalita G., Sharma S., Wakita K., Umeno M., Hayashi Y., Tanemura H. (2012). Synthesis of graphene by surface wave plasma chemical vapor deposition from camphor. Phys. Status Solidi A.

[32] Somani P.R., Somani P.S., Umeno M. (2006). Planer nano-graphenes from camphor by CVD. Chem. Phys. Lett..

[33] Ravani F., Papagelis K., Dracopoulos V., Parthenios J., Dassios K.G., Siokou A., Galiotis C. (2013). Graphene production by dissociation of camphor molecules on nickel substrate. Thin Solid Films.

[34] Chaliyawala H.A., Rajaram N., Patel R., Ray A., Mukhopadhyay I. (2019). Controlled island formation of large-area graphene sheets by atmospheric chemical vapor deposition: Role of natural camphor. ACS Omega.

[35] Chen W., Gui X., Yang L., Zhu H., Tang Z. (2019). Wrinkling of two-dimensional materials: Methods, properties and applications. Nanoscale Horiz..

[36] Ferrari A.C., Meyer J.C., Scardaci V., Casiraghi C., Lazzeri M., Mauri F., Piscanec S., Jiang D., Novoselov K.S., Roth S. (2006). Raman spectrum of graphene and graphene layers. Phys. Rev. Lett..

[37] Ferrari A.C. (2007). Raman spectroscopy of graphene and graphite: Disorder, electron–phonon coupling, doping and nonadiabatic effects. Solid State Commun..

[38] Graf D., Molitor F., Ensslin K., Stampfer C., Jungen A., Hierold C., Wirtz L. (2007). Spatially resolved Raman spectroscopy of single- and few-layer graphene. Nano Lett..

[39] Zhao P., Kim S., Chen X., Einarsson E., Wang M., Song Y., Wang H., Chiashi S., Xiang R., Maruyama S. (2014). Equilibrium chemical vapor deposition growth of Bernal-stacked bilayer graphene. ACS Nano.

[40] Xu X., Lin C., Fu R., Wang S., Pan R., Chen G., Shen Q., Liu C., Guo X., Wang Y. (2016). A simple method to tune graphene growth between monolayer and bilayer. AIP Adv..

[41] Li M., Liu D., Wei D., Song X., Wei D., Wee A.T.S. (2016). Controllable synthesis of graphene by plasma-enhanced chemical vapor deposition and its related applications. Adv. Sci..

[42] Hasegawa M., Tsugawa K., Kato R., Koga Y., Ishihara M., Yamada T., Okigawa Y. (2017). High quality and large-area graphene synthesis with a high growth rate using plasma-enhanced CVD. Synthesiology.

[43] Börrnert F., Barreiro A., Wolf D., Katsnelso M.I., Büchner B., Vandersypen L.M.K., Rümmeli M.H. (2012). Lattice expansion in seamless bilayer graphene constrictions at high bias. Nano Lett..

[44] Sze S.M. (1981). Physics of Semiconductor Devices.

[45] Zheng J., Wang L., Quhe R., Liu Q., Li H., Yu D., Mei W.-N., Shi J., Gao Z., Lu J. (2013). Sub-10 nm gate length graphene transistors: Operating at terahertz frequencies with current Saturation. Sci. Rep..

[46] Luongo G., Bartolomeo A.D., Giubileo F., Chavarin C.A., Wenger C. (2018). Electronic properties of graphene/p-silicon Schottky junction. J. Phys. D Appl. Phys..

[47] Tsai D.S., Liu K.-K., Lien D.-H., Tsai M.-L., Kang C.-F., Lin C.-A., Li L.-J., He J.-H. (2013). Few-layer MoS_2_ with high broadband photogain and fast optical switching for use in harsh environments. ACS Nano.

[48] Ghods A., Saravade V.G., Zhou C., Ferguson I.T. (2020). Field-effect passivation of metal/n-GaAs Schottky junction solar cells using atomic layer deposited Al_2_O_3_/ZnO ultrathin films. J. Vac. Sci. Technol. A.

[49] Periyanagounder D., Gnanasekar P., Varadhan P., He J.-H. (2018). Kulandaivel, High performance, self-powered photodetectors based on a graphene/silicon Schottky junction diode. J. Mater. Chem. C.

[50] Zhu M., Li X., Guo Y., Li X., Sun P., Zang X., Wang K., Zhong M., Wud D., Zhu H. (2014). Vertical junction photodetectors based on reduced graphene oxide/silicon Schottky diodes. Nanoscale.

[51] Gong X., Tong M., Xia Y., Cai W., Moon J.S., Cao Y., Yu G., Shieh C.L., Nilsson B., Heeger A.J. (2009). High-detectivity polymer photodetectors with spectral response from 300 to 1450 nm. Science.

[52] Xu H., Han X., Dai X., Liu W., Wu J., Zhu J., Kim D., Zou G., Sablon K.A., Sergeev A. (2018). High detectivity and transparent few-layer MoS_2_/glassy-graphene heterostructure photodetectors. Adv. Mater..

[53] An X., Liu F., Jung Y.J., Swastik Kar S. (2013). Tunable graphene–silicon heterojunctions for ultrasensitive photodetection. Nano Lett..

[54] Bartolomeo A.D., Giubileo F., Luongo G., Iemmo L., Martucciello N., Niu G., Fraschke M., Skibitzki O., Schroeder T., Lupina G. (2017). Tunable Schottky barrier and high responsivity in graphene/Si-nanotip optoelectronic device. 2D Mater..

[55] Lee M.-H., Cho Y., Byun K.-E., Shin K.W., Nam S.-G., Kim C., Kim H., Han S.-A., Kim S.-W., Shin H.-J. (2018). Two-dimensional materials inserted at the metal/semiconductor interface: Attractive candidates for semiconductor device contacts. Nano Lett..

[56] Mukherjee A., Yun H., Shin D.H., Nam J., Munshi A.M., Dheeraj D.L., Fimland B.-O., Weman H., Kim K.S., Lee S.W. (2019). Single GaAs nanowire/graphene hybrid devices fabricated by a position-controlled microtransfer and an imprinting technique for an embedded structure. ACS Appl. Mater. Interfaces.

[57] Shawkat M.S., Chung H.-S., Dev D., Das S., Roy T., Jung Y. (2019). Two-dimensional/three-dimensional Schottky junction photovoltaic devices realized by the direct CVD growth of vdW 2D PtSe_2_ layers on silicon. ACS Appl. Mater. Interfaces.

[58] Niu G., Capellini G., Lupina G., Niermann T., Salvalaglio M., Marzegalli A., Schubert M.A., Zaumseil P., Krause H.M., Skibitzki O. (2016). Photodetection in hybrid single-layer graphene/fully coherent germanium island nanostructures selectively grown on silicon nanotip patterns. ACS Appl. Mater. Interfaces.

[59] Kazemi I., Vaziri S., Morales J.D.A., Frégonèse S., Cavallo F., Zamiri M., Dawson N., Artyushkova K., Jiang Y.B., Brueck S.J.R. (2017). Vertical charge transfer and lateral transport in graphene/germanium heterostructures. ACS Appl. Mater. Interfaces.

[60] Shehzad K., Shi T., Qadir A., Wan X., Guo H., Ali A., Xuan W., Xu H., Gu Z., Peng X. (2017). Designing an efficient multimode environmental sensor based on graphene-silicon heterojunction. Adv. Mater. Technol..

[61] Lee D., Park H., Han S.D., Kim S.H., Huh W., Lee J.Y., Kim Y.S., Park M.J., Park W.I., Kang C.-Y. (2019). Self-powered chemical sensing driven by graphene-based photovoltaic heterojunctions with chemically tunable built-in potentials. Small.

[62] Pea M., Seta M.D., Gaspare L.D., Persichetti L., Scaparro A.M., Miseikis V., Coletti C., Notargiacomo A. (2019). Submicron size Schottky junctions on as-grown monolayer epitaxial graphene on Ge(100): A low-invasive scanned-probe-based study. ACS Appl. Mater. Interfaces.

[63] Abdalrheem R., Yam F.K., Ibrahim A.R., Lim H.S., Beh K.P., Ahmed A.A., Oglat A.A., Chahrour K.M., Farhat O.F., Afzal N. (2019). Improvement in photodetection characteristics of graphene/p-Silicon heterojunction photodetector by PMMA/graphene cladding layer. J. Electron. Mater..

[64] Tsai D.-S., Lien W.-C., Lien D.-H., Chen K.-M., Tsai M.-L., Senesky D.G., Yu Y.-C., Pisano A.P., He J.H. (2013). Solar-blind photodetectors for harsh electronics. Sci. Rep..

[65] Duan L., He F., Tian Y., Sun B., Fan J., Yu X., Ni L., Zhang Y., Chen Y., Zhang W. (2017). Fabrication of self-powered fast-response ultraviolet photodetectors based on graphene/ZnO:Al nanorod-array-film structure with stable Schottky barrier. ACS Appl. Mater. Interfaces.

[66] Wang G., Zhang M., Chen D., Guo Q., Feng X., Niu T., Liu X., Li A., Lai J., Sun D. (2018). Seamless lateral graphene p–n junctions formed by selective in situ doping for high-performance photodetectors. Nat. Commun..

[67] Fukushima S., Shimatani M., Okuda S., Ogawa S., Kanai Y., Ono T., Inoue K., Matsumoto K. (2019). Low dark current and high-responsivity graphene mid-infrared photodetectors using amplification of injected photo-carriers by photo-gating. Opt. Lett..

[68] Nie B., Hu J.-G., Luo L.-B., Xie C., Zeng L.-H., Lv P., Li F.-Z., Jie J.-S., Feng M., Wu C.-Y. (2013). Monolayer graphene film on ZnO nanorod array for high-performance Schottky junction ultraviolet photodetectors. Small.

